# Salient Materia Medica

**Published:** 1888-05

**Authors:** 


### BOOK NOTICES AND REVIEWS.

Salient Materia Medica and Therapeutics. By C. L. Cleveland, M. D. Pp. 171. Hahnemann Publishing House. F. E. Bœricke, Philadelphia, 1888.

One hundred and ninety-seven remedies are treated of in this little work, but they are considered in such a condensed manner as to be wholly useless.

